# A Novel Method for Regional NO_2_ Concentration Prediction Using Discrete Wavelet Transform and an LSTM Network

**DOI:** 10.1155/2021/6631614

**Published:** 2021-04-07

**Authors:** Bingchun Liu, Lei Zhang, Qingshan Wang, Jiali Chen

**Affiliations:** ^1^School of Management, Tianjin University of Technology, Tianjin 300384, China; ^2^School of Humanities, Tianjin Agricultural University, Tianjin 300384, China

## Abstract

Achieving accurate predictions of urban NO_2_ concentration is essential for effectively control of air pollution. This paper selected the concentration of NO_2_ in Tianjin as the research object, concentrating predicting model based on Discrete Wavelet Transform and Long- and Short-Term Memory network (DWT-LSTM) for predicting daily average NO_2_ concentration. Five major atmospheric pollutants, key meteorological data, and historical data were selected as the input indexes, realizing the effective prediction of NO_2_ concentration in the next day. Firstly, the input data were decomposed by Discrete Wavelet Transform to increase the data dimension. Furthermore, the LSTM network model was used to learn the features of the decomposed data. Ultimately, Support Vector Regression (SVR), Gated Regression Unit (GRU), and single LSTM model were selected as comparison models, and each performance was evaluated by the Mean Absolute Percentage Error (MAPE). The results show that the DWT-LSTM model constructed in this paper can improve the accuracy and generalization ability of data mining by decomposing the input data into multiple components. Compared with the other three methods, the model structure is more suitable for predicting NO_2_ concentration in Tianjin.

## 1. Introduction

With the development of urbanization and industrialization, per capita energy consumption increases year by year. In addition to natural sources such as dust storms, bush fires, and volcanic eruptions, increased nitrogen dioxide (NO_2_) from vehicle exhaust and boiler exhaust has become one of the major environmental problems facing most countries in the world [1]. In China, the number of air quality standards and the average number of days with good air quality in 338 cities have increased year by year recently. However, in the Beijing-Tianjin-Hebei region and surrounding areas, the concentration of six air pollutants (PM2.5, PM10, O_3_, SO_2_, NO_2_, and CO) decreased the least, except ozone (O_3_). And, the same is true for Tianjin. In addition, the annual average concentration of NO_2_ in Tianjin in 2018 was 47 micrograms per cubic meter, exceeding the national annual average concentration standard (40 micrograms per cubic meter). NO_2_ has become the most important pollutant affecting the air quality of Tianjin. All of these data above are obtained from the website http://www.mee.gov.cn/hjzl/zghjzkgb/lnzghjzkgb/.

In fact, nitrogen dioxide dissolves in water in the air to form acids, which may lead to the occurrence of acid rain and react with ultraviolet radiation to form photochemical smog. In addition, human exposure to NO_2_ of different concentrations may lead to lung function damage of different degrees, seriously affecting industrial production and social activities [2]. Therefore, the effective detection and accurate prediction of NO_2_ and the establishment of a highly accurate and stable prediction model can provide an early warning of air pollution emergencies and guide the release of NO_2_ control measures and public health protection work.

The existing research on the prediction of atmospheric pollutant concentration can be roughly divided into three categories. The first category is the deterministic method based on the physical and chemical change model of the atmosphere [3–5]. The second category is the use of computational methods based on regression and neural networks. The third category is the optimal combination model based on the second category. The deterministic approaches with no need for a large amount of historical data require a complete knowledge of pollution sources, timely emission quantities, main chemical reactions of the gaseous pollutants, and spatiotemporal physical transformation processes. The second and third types of computational methods usually require a large amount of historical measurement data under various meteorological conditions.

Wang et al. [6] used the Weather Research and Forecasting model coupled with Chemistry (WRF—Chem) for a serious pollution incident in Beijing in December 2016. The accompanying sensitivity analysis in this paper could capture the influence of emission sources on the concentration of target pollutants in different regions and different time periods, which provided a good reference for formulating effective emission reduction measures and regional air pollution prevention and control. However, due to the limitations of the resolution of existing models and other issues, the pollution events studied cannot be further utilized for other pollution events of different seasons and types. Baykara et al. [7] applied CMAQ (5.2), based on the heating activity data of local residents in Istanbul, to explore the influence of emissions from the residential heating sector on the level of environmental particulate matter. They thought that winter was the time when residential heating sector mainly affects regional air quality. A possible reason for this was the increase in coal burning that produces sulfur dioxide emissions but not for other man-made emissions.

The data-driven model mainly conducts a statistical analysis of air quality data and related factors to obtain scientific conclusions. In terms of linear correlation analysis, scholars have put forward a series of methods, such as geographic weighted regression (GWR), geographical and time-weighted regression (GTWR), and land use regression (LUR). Hinojosa-Baliño et al. [8] used meteorological, demographic, geographical, and social data and mixed geographic information system (GIS) and LUR to generate the prediction model and spatial distribution of PM2.5 air pollution. Alahmadi et al. [9] used a local GWR model in the GIS environment to describe and quantify the contribution of transportation sector emissions to the NO_2_ concentration in the Red Sea, but the limitation is the unavailability of some data. Further, Mirzaei et al. [10] used the GTWR model to study the spatial-temporal variability between PM2.5 concentration at ground monitoring stations and satellite aerosol optical depth (AOD) data. However, in warm season, there were defects in the retrieval algorithm when detecting the low value of particulate matter, which lead to a decrease in the prediction accuracy of the model, and thus affected the simulation output. The application of machine learning algorithms, such as random forests, support vector machines (SVMs), and artificial neural networks (ANNs), takes the nonlinear relationship into consideration and improves the accuracy of prediction [11–13]. Masih [14] employed an integrated data mining tool that used random forests to predict the concentration of nitrogen dioxide in the atmosphere taking the emission inventory and meteorological parameter monitoring data set as input prediction factors, and compared them with M5P and SVM, demonstrating the superiority of the model. Liu et al. [15] used support vector regression (SVR) to make a collaborative prediction of the Chinese urban air quality index (AQI). Experiments showed that when there was a strong interaction and correlation between air quality characteristic attributes and the air quality index, the MAPE (Mean Absolute Percentage Error) value of the multicity multidimensional regression model decreased. Cabaneros et al. [16] applied a mixed artificial neural network to the prediction of urban road NO_2_. Mishra and Goyal [17] developed an NO_2_ concentration prediction model based on an artificial intelligence neuro-fuzzy model. However, these prediction models cannot capture both long-term and short-term characteristics, so Long Short-Term Memory (LSTM) is often used to predict air quality and pollutant concentration with time series characteristics. Li et al. [18] used LSTM layers to automatically extract inherent useful features of atmospheric pollutant data to predict the PM2.5 concentration in Beijing in the next hour. Following this, Reddy et al. [19] extended the prediction from a single time step to the next 5 to 10 hours based on the time series data of pollution and meteorological information of Beijing.

However, the above model may lead to insufficient accuracy due to its potential convergence to local minima and overfitting [20]. In recent years, with the development of artificial intelligence and big data analysis, hybrid methods based on various information processing methods and deep learning methods have been widely used [21–26]. Kordestani and Samadi used distributed neural network and Bayesian algorithm to predict the remaining service life of Multifunctional Spoilers (MFS). Taking the data of LJ2000 series fighter data as samples, the hybrid prediction method was evaluated with relative accuracy, and it was found that the prediction effect of distributed structure was better [27]. Rezamand et al. constructed a hybrid prediction method based on real-time Supervisory Control and Data Acquisition (SCADA) and vibration signals to predict the Remaining Useful Life (RUL) of wind turbine bearings, made an empirical analysis of the hybrid model, and concluded that the prediction accuracy of this method was higher than that of the Bayesian algorithm [28]. Chen, Zhang, and Vachtsevanos proposed a prediction method of machine health condition based on Neural-Fuzzy Systems (NFSs) and Bayesian algorithm. Two examples of a cracked bearing plate and a faulty bearing were used to verify the effectiveness of the hybrid prediction method. The experimental results show that the hybrid method can effectively predict the running condition of the machine [29]. Bai et al. [30] proposed a neural network with long- and short-term memory (E-LSTM) to predict PM2.5 concentration per hour and added mode decomposition (EMD) to the LSTM foundation, effectively improving the prediction accuracy. Zhao et al. [31] proposed a data-driven model called the LSTM-FC neural network, which uses historical air quality data, meteorological data, and weather forecast data to predict PM2.5 pollution over 48 hours at a particular air quality monitoring station. Other researchers, such as Pak et al. [32], proposed that a mixed model convolutional neural network (CNN) combined with long- and short-term memory (CNN-LSTM) has better seasonal stability and prediction performance compared with the single LSTM model. Wu and Lin [33] developed a hybrid model called VMD-SE-LSTM which applies VMD (Variational Mode Decomposition) technique to decompose the AQI data and employed SE (Sample Entropy) to recombine these components and train each recombinant subsequence with an LSTM neural network. This model not only improves the precision but also has good generalization ability.

Although the data decomposition and deep learning model have been combined in existing studies, the combination model of wavelet transform and deep learning method has not been applied to the prediction of air pollutant concentration. The novelties of this study is to take Tianjin as an example and build a DWT-LSTM combined model to predict the NO_2_ concentration of the city in the future. In addition, through an empirical analysis of the NO_2_ concentration in Tianjin, it is concluded that the prediction effect of the DWT-LSTM model constructed in this paper is better than that of the SVR, GRU, and single LSTM models. At the same time, the prediction results can provide early warning for air pollution emergencies in Tianjin and guide the introduction of NO_2_ control measures and public health protection.

The above research is of certain use for the prediction of NO_2_ concentration in the regional atmosphere. In this study, a data processing method based on discrete wavelet decomposition combined with an LSTM deep learning algorithm achieves the purpose of relatively accurate prediction of NO_2_ concentration in Tianjin. The main contributions of this study are as follows: (1) the use of wavelet decomposition to carry out dimensional processing of data, optimize input variables, and improve the prediction accuracy of the LSTM model; (2) the development of the DWT-LSTM prediction model; (3) the consideration of the correlation between traditional LSTM prediction results and wavelet-LSTM results and actual data, verifying that data can improve the prediction accuracy and stability of the LSTM model through wavelet decomposition.

## 2. Research Area and Data

**Figure 1:**
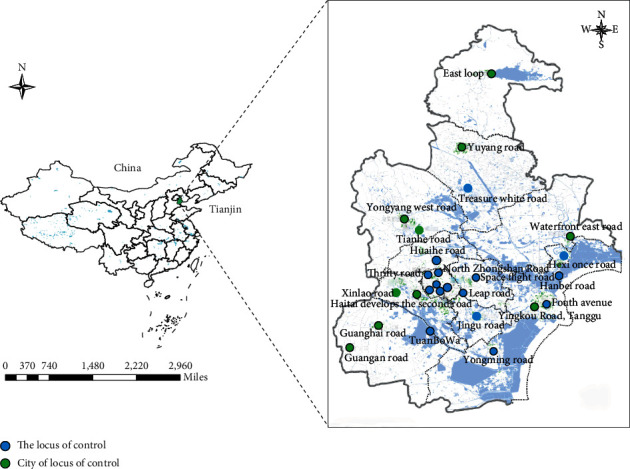
Location of the environmental monitoring stations.

The geographical area of this study is Tianjin, China, located in the north China plain (117:10e39:10n, Figure 1). As of July 1, 2019, the Tianjin environmental air quality monitoring network has been established, covering the central urban area, the four districts around the city, the new Binhai area and other districts. Each point has six regular air pollutant monitoring capabilities, including PM10, PM2.5, SO_2_, NO_2_, CO, and O_3_. Since 2013, the Tianjin environmental air quality GIS platform has been used to release environmental air quality information for all monitoring points in Tianjin to the public. Now there are 16 national control stations and 11 municipal control stations with a total of 27 testing stations.

We collected the daily average data of PM2.5, PM10, NO_2_, SO_2_, O_3_, and CO on January 1, 2014, solstice to June 30, 2019 (2007 days) in Tianjin and used the latest available data to correct the missing data of each type of air pollutant. In addition, we also downloaded meteorological observation data from the Chinese meteorological website platform established by the China meteorological administration (CMA), including wind speed, temperature, and weather conditions.

**Figure 2:**
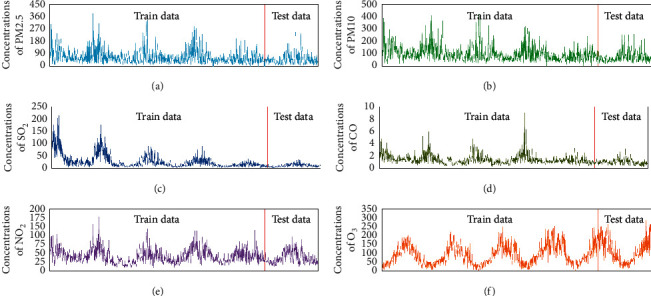
Daily mean concentration of 5 air pollutants and NO_2_ concentration from the previous day during the period 1/11/2014–30/6/2019.

The output predictor of the experiment is the daily average concentration of NO_2_ in Tianjin, which is shown in Figure 2. In Figure 2(e), it can be seen that the NO_2_ concentration exhibits obvious periodicity, namely, high concentrations in the winter and summer concentration is low. Figures 2(a)–2(d) show the graph of the concentrations of PM2.5, PM10, SO_2_, and CO, respectively, over the same period. It can be seen that the four kinds of pollutant and NO_2_ exhibit the same periodic variation. Figure 2(f) shows the O_3_ concentration graph, where it can be seen that O_3_ has periodic changes that are opposite to the other five pollutants. This may be because low-altitude O_3_ is usually prone to produce and exceeds the standard in high temperature seasons [34]. The wind force was quantified according to the method of Bai et al. [35]. We graded the weather conditions according to how good or bad they were and quantified the weather indicators. The data (five pollutants, temperature, weather, wind data, and historical NO_2_) from January 2, 2014, to May 26, 2018, were used for training. The data from May 27, 2018, to June 30, 2019, were used for testing, also in conjunction with the NO_2_ historical data.

Statistical descriptions of six pollutants are given in Table 1, where O_3_ is the 8-hour average concentration.

## 3. Methodologies

### 3.1. Long Short-Term Memory

The LSTM neural network is a popular recursive neural network algorithm, which was first proposed by Hochreite and Schmidhuber to improve the memory of long (static) and short (cyclic) dynamic features of time series [36]. Similar to the traditional recurrent neural network model, this approach models time data by mining the circular connections between neurons and mining the internal connections between time series data. However, unlike traditional circular neural network models, it has a unique neuron structure called a “memory unit.” The hidden layer of an LSTM network constructed by this approach can store time information of any length to obtain a more accurate time series model.

**Figure 3:**
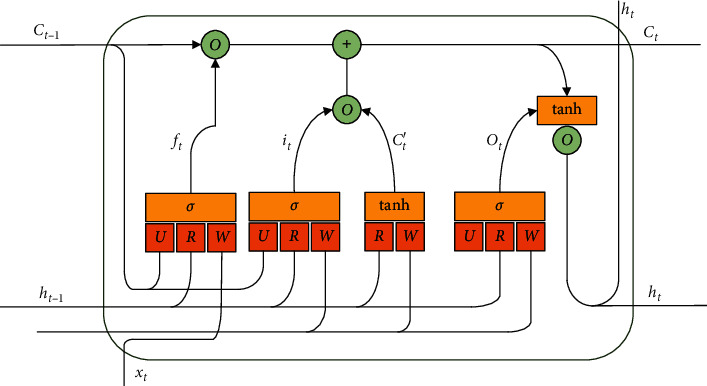
Structure of an LSTM neural network.

The memory unit structure of the LSTM network is shown in Figure 3 [37]. The fixed length window of the time series is generated and input into the LSTM network. Multiple LSTMs can be superimposed to learn more complex patterns of sequential information [38]. The memory module consists of an input gate, forgetting gate, output gate, and a loop unit. Its core idea is to control the switch of each gate by a nonlinear function, to protect and control the state of the memory unit, so as to control the increase or decrease of information [39]. Therefore, the key of an LSTM network is to store data information through the state of the storage unit for a long time. In general, the output value of the three gates is 0∼1, and the sigmoid function is used to determine how much information can be input to the memory location. The main formula is as follows:

(1) $$\begin{array}{c} i_{t}=\sigma \left(W^{i}x_{t}+R^{i}h_{t-1}+U^{i}\circ c_{t-1}+b^{i}\right), \end{array}$$

(2) $$\begin{array}{c} f_{t}=\sigma \left(W^{f}x_{t}+R^{f}h_{t-1}+U^{i}\circ c_{t-1}+b^{f}\right), \end{array}$$

(3) $$\begin{array}{c} c_{t}^{\prime }=\tanh\left(W^{c}x_{t}+R^{c}h_{t-1}+b^{c}\right), \end{array}$$

(4) $$\begin{array}{c} c_{t}=f_{t}\circ c_{t-1}+i_{t}\circ c_{t}^{\prime }, \end{array}$$

(5) $$\begin{array}{c} o_{t}=\sigma \left(W^{o}x_{t}+R^{o}h_{t-1}+U^{o}\circ c_{t}+b^{o}\right), \end{array}$$

(6) $$\begin{array}{c} h_{t}=o_{t}\tanh\left(c_{t}\right), \end{array}$$

where *o* represents the Hadamard product and tanh is used as the activation function. *x*_*t*_, *c*_*t*_, and *h*_*t*_ are the input, storage unit state, and output of the LSTM at time *t*, respectively, while *i*_*t*_, *o*_*t*_, and *f*_*t*_ are the function values of the input gate, output gate, and forgetting gate, respectively. *c*_*t*_′ is the input modulate gate, which determines how much new information can be received. *σ*(·) is the sigmoid function, *R*, *W*, and *U* are weight matrixes, *i*is the input, *f* is the forget, *c* is the cell structure, *o* is the output. *W*_*s*_ represents the weight matrices, and its superscript represents the two variables connected by the matrix. For example, *W*^*i*^ is the weight matrix between the input and the input gate and *b* is the deviation of the gate.

### 3.2. Discrete Wavelet Transform

Considering that the concentration of NO_2_ in the atmosphere is related to the other five major pollutants, wind force, weather conditions, temperature difference, and other factors, the daily average NO_2_ concentration series is nonstationary, volatile, and time-ordered. The above factors have different effects on NO_2_ concentration. The input signal contains various frequency components: the contributions of low-frequency and high-frequency components to the dynamic characteristics of wind power data are different. If the components of these different frequencies can be learned by independent LSTMs, it will improve the performance of data mining. Therefore, the divide and conquer strategy requires that the original wind power data be decomposed into low-frequency and high-frequency signals through appropriate decomposition algorithms. In this paper, a discrete wavelet transform is used to decompose the original input data. It makes use of the time scale function to analyze the data and makes the wavelet transform have a multiscale resolution and time-shift characteristics. The scaling operation can observe signals of different scales. Therefore, a wavelet transform is very suitable for dealing with nonstationary time series including air pollutant data.

Assuming that *x*(*t*) squared can be integrated, *x*(*t*) can be expanded under the wavelet basis function. This operation is called the continuous wavelet transform of *x*(*t*). The mathematical definition of the wavelet basis function is

(7) $$\begin{array}{c} \Psi_{a,b}\left(t\right)=\frac{1}{\sqrt{\left|a\right|}}\Phi \left(\frac{t-b}{a}\right),\;a\neq 0,b\in R. \end{array}$$

The mathematical definition of the *x*(*t*) continuous wavelet transform is given as

(8) $$\begin{array}{c} W_{x}\left(a,b\right)=\langle x\left(t\right),\Psi_{a,b}\left(t\right)\rangle =\frac{1}{\sqrt{\left|a\right|}}\int_{R}x_{t}\left(t\right)\bar{\Phi \left(\frac{t-b}{a}\right)}\text{d}t. \end{array}$$

In equations (7) and (8), *ψ*(*t*) can be considered as the parent wavelet function. *a* is the scale parameter and *b* is the time center parameter. When *a* and *b* change continuously, the whole transformation process is called a continuous wavelet transform. However, in practical applications, continuous transformation greatly increases the computational complexity, application cost, and implementation difficulty and is usually replaced by a small step discrete wavelet (DWT) [40].

A discrete wavelet transform makes the application of a wavelet transform easy to realize. The exponential discretization of parameters *a* and *b* reduces the computational complexity and avoids the information redundancy brought by a continuous wavelet transform. The discrete wavelet transform of *x*(*t*) is defined as

(9) $$\begin{array}{c} W_{x}\left(j,k\right)=\langle x\left(t\right),\Psi_{j,k}\left(t\right)\rangle =\frac{1}{\sqrt{\left|a\right|}}\int_{R}x_{t}\left(t\right)\bar{\Phi \left(\frac{t-b}{a}\right)}\text{d}t. \end{array}$$

In equation (9), *a* and *b* are discrete. *a*=*a*_0_^*j*^, *b*=*ka*_0_^*j*^*b*_0_, *a*_0_ > 1, *b*_0_ > 0, *j* ∈ *Z*, *k* ∈ *Z*.

The discrete wavelet transform is the Mallat algorithm proposed in 1988 [41]. This is actually a signal decomposition method. For the multiresolution characteristics of wavelets, variable *j* is used to determine the resolution at different scales. Specifically, the main outline of the original signal is observed on a large scale, and the detailed information of the original signal is observed on a small scale. Finally, with gradually increasing *j* the results come out: one approximation value (i.e., low-frequency component) and *n* (which needs to be artificially set) detailed signals (i.e., high-frequency) *d*_*n*_, *d*_*n*−1_, *d*_*n*−2_,…, *d*_1_ [42]. The original signal and two kinds of subsignals are satisfied by the following formula:

(10) $$\begin{array}{c} x\left(t\right)=a_{n}+d_{n}+d_{n-1}+\cdots +d_{1}. \end{array}$$

**Figure 4:**
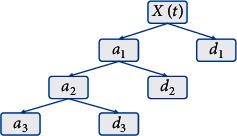
Three-layer wavelet decomposition.

The schematic diagram of the discrete wavelet transform decomposition is shown in Figure 4.

### 3.3. Overview of DWT-LSTM

**Figure 5:**
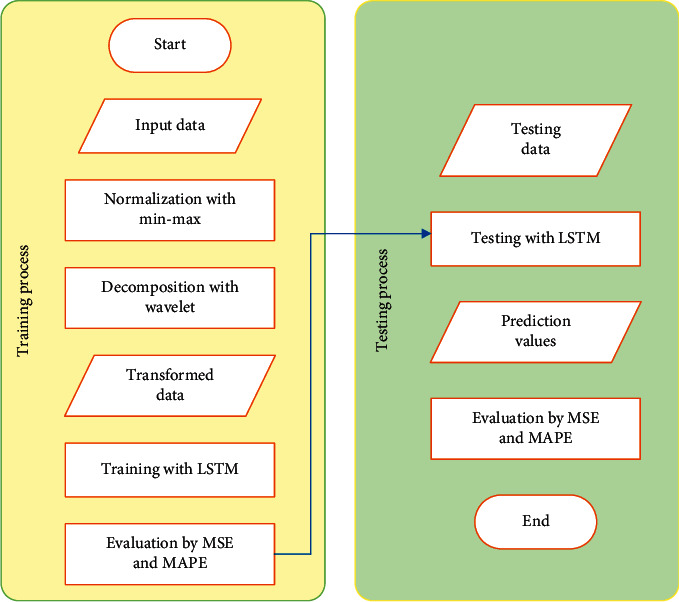
Prediction experiment process.

The LSTM neural network model is used to identify data patterns, and wavelet decomposition is used to decompose the input data. The prediction model combined with the wavelet transform and LSTM neural network consists of the following stages:

  Step 1: add the original data set {*M*_1_, *M*_2_,…, *M*_*n*_} which is normalized, and the experimental data set {*D*_1_, *D*_2_,…, *D*_*n*_};
  Step 2: set {*D*_1_, *D*_2_,…, *D*_*n*_} can be decomposed through *m* layers to obtain the high-dimensional input information set {*X*_1_′, *X*_2_′,…, *X*_*t*_′}, where *X*_*i*_′=(*A*_*mi*−1_, *D*_*li*−1_,…, *D*_*mi*−1_), *I* = 1,2,…, *t*. The decomposition result at time *t* + 1 is *X*_*t*+1_′=(*A*_*mt*_, *D*_*lt*_,…, *D*_*mt*_);
  Step 3: use the new data set {(*X*_*i*_′, *Y*_*i*_)}_*i*=1_^*t*^ to train the LSTM model. Through repeated data training and data testing, adjust parameters and get the optimal prediction model *f*(*X*_*i*_), as shown in Figure 5.
  Step 4: the predicted value *f*(*X*_*t*+1_′) of the concentration of air pollutants in stage *t*+1 can be measured by using the prediction model obtained above and according to the input vector *X*_*t*+1_ obtained in stage *t*+1.
  Step 5: repeat steps 1–4 to obtain the predicted results *f*(*X*_1_′),…, *f*(*X*_*t*+1_′).

As the purpose of this study is to predict the Tianjin daily average concentration of NO_2_, the input index includes two kinds of data, pollutant concentration, and meteorological factors. First, the meteorological factor information is quantified for further processing. The quantitative data are used for integration with other numeric data, but due to abnormal fluctuations, it will seriously affect the prediction ability. The consolidated data are normalized using Min-Max methods [43]:

(11) $$\begin{array}{c} d_{ij}=\frac{m_{ij}-\text{Min}\left(m_{i1},m_{i2007}\right)}{\text{Max}\left(m_{i1},m_{i2007}\right)-\text{Min}\left(m_{i1},m_{i2007}\right)},\;i=1,2,\ldots ,9;j=1,2,\ldots ,2007. \end{array}$$

**Figure 6:**
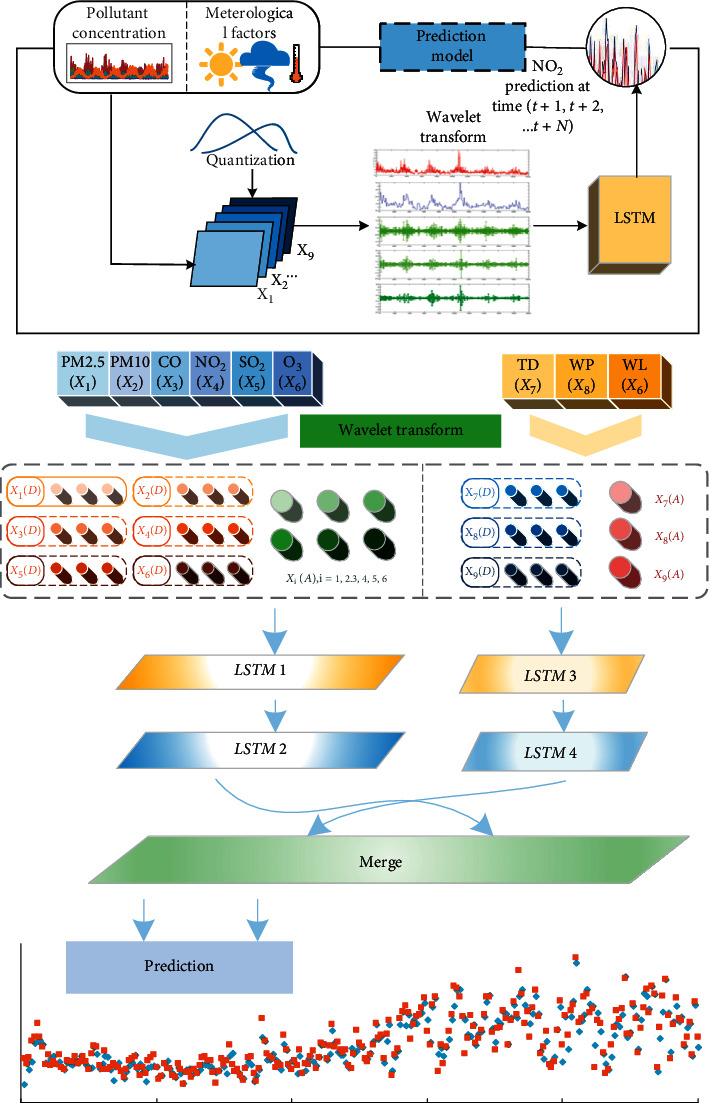
Architecture of the LSTM network with discrete wavelet transform (DWT-LSTM).

The normalized data are used as the original signal for wavelet decomposition. As shown in Figure 6, a group of low-frequency subsignals and three groups of high-frequency subsignals of each original data were selected as input data and used for training and validation by LSTM neural networks. The best prediction model is obtained by adjusting the parameters and the structure of the design model.

### 3.4. Model Parameters and Performance Indicator

The prediction model proposed in this paper was implemented using *Python* 2.7 in Matlab 2017a and the Linux system environment. The DWT-LSTM model adopts 3-layer wavelet decomposition and the Daubechies (DB) wavelet basis function. In network parameter setting, the primary parameters of LSTM include learning rate, max epochs, batch size, number of hidden layers, and tine step. In the best model, learning rate is 0.0001, max epoch is taken as 500, number of hidden layers is 32, and time step is 3. The selection of the relevant parameters in the model targets mean absolute percentage error (MAPE) minimization [44], and this is an important indicator to measure prediction accuracy in the statistical field and is also widely used in the prediction of air pollutant concentrations. The MAPE index was used to measure the error of the prediction algorithm and compare it with other algorithms. Not only was the error between the predicted value and the true value considered but also the ratio between the error and the true value was considered [45]. The following equation gives the calculation of MAPE:

(12) $$\begin{array}{c} \text{MAPE}=\frac{1}{n}\sum_{i=1}^{n}\frac{\left|y_{i}-y_{i}^{*}\right|}{y_{i}^{*}}, \end{array}$$

where *y*_*i*_^*∗*^ is the observed NO_2_ concentration, *y*_*i*_ is the predicted NO_2_ concentration, and *n* is the number of detected samples.

## 4. Results and Discussion

### 4.1. Data Description

This paper selects six air pollutants of PM2.5, PM10, NO_2_, SO_2_, O_3_, and CO and three meteorological observation factors of wind speed, maximum temperature, and minimum temperature, as input indexes. In order to evaluate the accuracy of the NO_2_ concentration prediction model, the index data from January 1, 2014 to June 30, 2019, with a total of 2007 points, were selected in this paper. The original data sample was divided into two data sets: 80% of the original data (1606 data points) were used as the training sample, and the remaining 20% of the original data (401 data points) were used as the test sample to evaluate the prediction performance of the model.

### 4.2. Results of the Wavelet Transform

The LSTM method is suitable for time series prediction as it has good prediction performance. Also, the LSTM model can effectively represent the nonlinear relationship between the input vector and prediction target through the use of a kernel function. Appropriate high-dimensional input vectors can describe the information in features more effectively and accurately and express the meaning of the data. Therefore, the prediction performance depends largely on the choice of input vector in model design. In this study, when the LSTM model is used to predict pollutant concentration, in order to make the prediction results more accurate and stable, the structural transformation of the input variables can be determined to obtain a new set of input variables. By using wavelet decomposition, the data are promoted from one-dimensional data to high-dimensional data, which fully represents the trend of data change and improves the prediction accuracy. In this study, wavelet decomposition is based on the wavelet basis function of Daubechies (DB). Daubechies has low-pass and high-pass filtering characteristics and is suitable for feature selection. Due to its inherent orthogonality, the Daubechies wavelet can be used widely and shows good performance in analyzing applied time series data.

**Figure 7:**
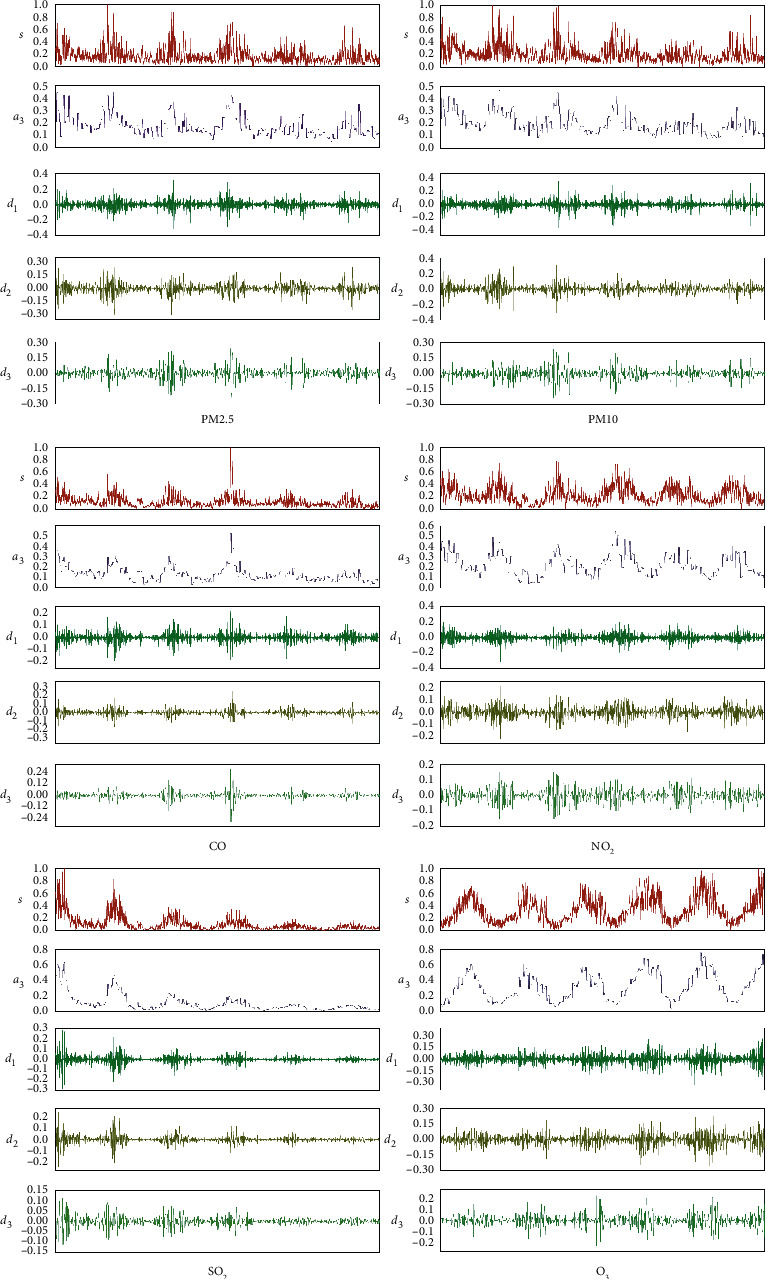
The result of the wavelet transform.

Using the Matlab tool, low-frequency approximate information and high-frequency information obtained by wavelet decomposition transformation are taken as another new input vector group of the LSTM model to form a new prediction data set of the six kinds of air pollutants (PM10, PM2.5, NO_2_, SO_2_, O_3_, and CO). The transformation results are provided in Figure 7, which shows the high-frequency information group and the low-frequency information group. The set of wavelet decomposition transforms the density time series data of the three input characteristic variables to generate high-dimensional input vectors, which effectively increases the amount of data representation information and significantly improves the prediction stability of the model.

### 4.3. Result of Prediction

We compared the performance of the proposed DWT-LSTM model with that of SVR, GRU and the single LSTM model and trained and tested these models with the same training and test set applicable to the DWT-LSTM model. In order to evaluate the effectiveness of this method, we added two indexes: root mean square error (RMSE) and average absolute error (MAE). These indicators can be expressed as follows:

(13) $$\begin{array}{c} \text{RMSE}=\sqrt{\frac{1}{n}\sum_{i=1}^{n}{\left(y_{i}-y_{i}^{*}\right)}^{2}}\text{,}\\ \text{MAE}=\frac{1}{n}\sum_{i=1}^{n}\left|y_{i}-y_{i}^{*}\right|. \end{array}$$

**Figure 8:**
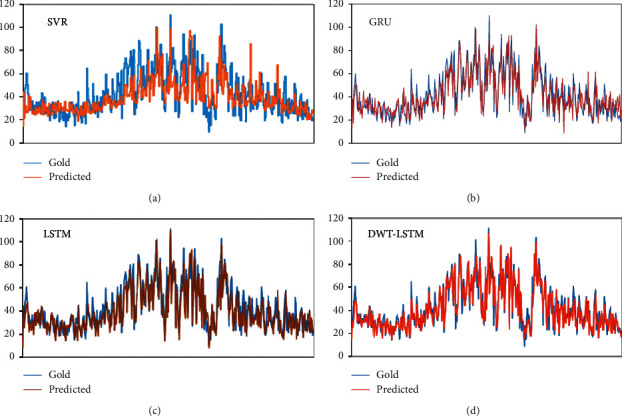
The forecasting results of different models.

Figure 8 intuitively shows the experimental results of the four prediction models. Through visual analysis, it can be seen that the prediction curve of the SVR model is relatively flat, it is difficult to accurately predict the fluctuation of data, and it presents a fluctuation trend opposite to the target value in some time periods. GRU, a variant of the LSTM, algorithmically combines forgetting and input gates into a single update gate, as well as a mixture of cellular and hidden states, and other changes. Although it has better performance in some experiments [46], its performance in this experiment is not as good as that of the single traditional LSTM model; especially it cannot predict outliers well. The LSTM model performs well regarding outliers (maxima and minima). For example, the prediction accuracy of a single LSTM model is better than that of the other three models on the two maxima of day 185 and day 206 and the two minima of day 155 and day 259. In order to objectively evaluate the performance of the four models, we calculated the predicted results according to the above formula, and the results are shown in Table 2. The evaluation results show that the performance of the DWT-LSTM model is better than that of the other three neural network models. Although the performance of the predicted outliers is not as good as that of the single LSTM model, the overall prediction accuracy is the highest.

The value of MAE and RMSE can explain the above phenomena, and the average absolute error and the average error of the LSTM model are greater than the DWT-LSTM model, which shows that the predictive value of the LSTM model is large, so it is also more likely to approximate the real value in the case of abnormal values. But the average absolute error and the average mean and mean error are relatively small, and the relatively small number of changes is far from the real value, which is higher than the other models and can more effectively predict the change of the concentration of NO_2_, which can be more effective in the prediction of other areas or other pollutants and more effectively guide the prevention and control of air pollution.

### 4.4. Analysis of Influencing Factors

In order to explore the relationship between various factors and NO_2_ concentration, we changed the input indexes and conducted a series of experiments:

1. To investigate whether the weather conditions, wind power, and temperature difference are related to the concentration of NO_2_, we eliminate the meteorological index and only used NO_2_ historical data and other 5 pollutants as input indicators for fitting. The results showed that the MAPE increased from 11.58% to 13.54%, proving that the meteorological index correlated with the concentration of air pollutants. Then, on the basis of the above experiments, we successively added weather condition indicators, temperature difference, and wind force in the experiments, and the MAPES were 13.63%, 13.61%, and 11.53%. It is proved that among the three meteorological factors, wind power has the largest effect on NO_2_ concentration; weather condition and temperature difference do not have much effect.
2. In order to explore the relationship between pollutant concentration and NO_2_ concentration, we firstly forecast the historical data of NO_2_ as a single input, and the MPAE is 16.61%. This proves that the historical data alone cannot effectively predict the future NO_2_ concentration. As a result, we will explore which major air pollutants have the greatest impact on NO_2_ in the future. On the basis of the historical data of NO_2_, we successively add PM2.5, PM10, SO_2_, CO, and O_3_ to carry out five groups of experiments, and the MAPE of the results is 14%, 14.96%, 15.39%, 12.68%, and 16.8%. It is shown that the effect of CO on NO_2_ concentration is the largest, followed by PM2.5 and PM10, SO_2_, and O_3_ having little effect on NO_2_ concentration.

### 4.5. Analysis of NO_2_ Concentration Change

From 2014 to the first half of 2019, the NO_2_ concentration in Tianjin showed an overall downward trend. The seasonal periodic change rule remained unchanged, and the peak value in winter also showed a downward trend every year. This shows that Tianjin has paid more attention to the ecological environment during the 13th five-year plan period, and its specific work has achieved results.

1. Since 2011, Tianjin has been working in the development of new energy vehicles and has issued a series of related documents. By 2015, energy saving and new energy vehicles in the city increased to a total of more than 60000. In 2017 and 2018, this increased to 79000 vehicles. The promotion of new energy vehicles resulted in 36% of the total transport buses being new energy buses, bringing the total that has been put into operation to 3670. By the end of April 2019, the number of new energy vehicles in the city had reached 125,000. As a result, the coal consumption and oil consumption in Tianjin also tended to decline, and the concentration of NO_2_ in the air also decreases accordingly.
2. During this period, Tianjin carried out the optimization and upgrading of traditional industries and forced the closure of a series of enterprises with serious pollution emissions, especially internal combustion engine production enterprises and nonferrous and ferrous metal smelters. On the basis of the traditional manufacturing industry, the industrial structure has been adjusted, focusing on the development of high-end equipment, new-generation information technology, aerospace, new energy vehicles, new materials, biomedicine, new energy, energy conservation and environmental protection, modern petrochemical, modern metallurgy, and ten other industries, while paying attention to the development of the service industry. Therefore, NO_*x*_ emissions are reduced by reducing emission sources.
3. We can clearly find that the concentration of NO_2_ in spring and winter is higher than that in summer and autumn in Tianjin. This phenomenon may be caused by the low temperature in spring and winter, which is not conducive to the diffusion of NO_2_ and leads to an increase in concentration. For Tianjin, this may be related to the cold weather in winter and spring, which requires a large amount of coal burning and some natural gas to heat the city. Fossil fuels such as coal produce a large amount of nitrogen dioxide, leading to an increase in the total NO_2_ content. In addition, although fireworks are banned in Tianjin, fireworks are still set off in rural areas due to insufficient supervision, leading to an increase in the concentration of NO_2_ in the city.

### 4.6. Cause of Prediction Deviation

In 2012, the ministry of environmental protection and the general administration of quality supervision, inspection, and quarantine jointly issued the environmental air quality standard (GB 3095-2012), which has been implemented nationwide since January 1, 2016. The implementation of the new standard is slightly different from that before in the way pollutants are counted, so the numerical performance is inconsistent within the statistical range. In addition, the accumulation of historical data is insufficient, the high concentration value of heavy pollution days is less, and the limited training data brings great uncertainty to the prediction of high values.

On the other hand, DWT-LSTM is a data-based statistical model, which mainly relies on the empirical formula of meteorological parameters and historical monitoring data, and fails to consider the atmospheric chemical transformation, pollution source emission change, and regional transmission process. NO_2_ reacts photochemically with O_3_ and acts as a catalyst in the air to convert O_2_ into O_3_. Most of the NO produced by human activities comes from the combustion of fossil fuels, such as automobiles, airplanes, internal combustion engines, and industrial kilns. It also comes from the process of producing and using nitric acid, such as nitrogenous fertilizer plant, organic intermediate plant, nonferrous, and ferrous metal smelting plant. NO reacts with the oxygen in the air in the atmosphere, generating NO_2_. Therefore, NO_2_ is associated with changes of industrial structure adjustment and pollution emissions. At the same time, external sources also contribute significantly to pollutant concentration during heavy pollution period.

## 5. Conclusions

In this study, a combined prediction model was established based on discrete wavelet decomposition and a neural network method to predict NO_2_ concentration in Tianjin. The conclusions are as follows:

1. The combined prediction model uses wavelets to decompose the time series data of air pollutant concentration and takes the low-frequency and high-frequency data obtained after decomposition as input variables at the same time, thus increasing data dimensionality. Through the use of information representation of pollutant concentration time series data at different frequencies, the characteristics of the data can be better described.
2. The prediction model was built using an LSTM neural network, a high-dimensional nonlinear learning algorithm. When applied to the prediction of Tianjin NO_2_ concentration, the performance was not as good as that of a single LSTM model, but the overall prediction accuracy was the highest. However, due to the low dimensionality of pollutant concentration time series data, the representation of the information is incomplete, affecting the ability of the prediction model to generalize.
3. The DWT-LSTM neural network method can be used to accurately predict the air pollutant concentration. Compared with the single LSTM model, the MAPE decreased from 17.85% to 11.58%; the MAE and RMSE increased to 4.3377 and 5.9291, respectively.
4. The practical significance of the use of a statistical prediction method for urban development and social activities is demonstrated by discussing the reasons underpinning prediction deviations and NO_2_ concentration change.

In the process of data modeling, because of the limitation of the hardware used in the experimentation, the data cannot be fully analyzed, including the exploration of the structure of the neural network, leading to less complex model design. Research in the future will increase the dimension of data collection and carry out further experiments and explorations under the improved hardware environment.

## Figures and Tables

**Table 1:** Statistical descriptions of the main indicators.

	PM_2.5_	PM_10_	SO_2_	CO	NO_2_	O_3_
Unit	*μ*g/m^3^	*μ*g/m^3^	*μ*g/m^3^	mg/m^3^	*μ*g/m^3^	*μ*g/m^3^
Mean	66.49	106.18	23.87	1.33	47.39	93.07
Std.	49.71	65.21	25.53	0.74	21.98	56.67
Min	13	38	6	0.6	19	10
Max	383	483	23	9	176	286

**Table 2:** Comparison of forecasting performances using different models.

Method	MAPE (%)	MAE	RMSE
SVR	25.8688	10.1734	13.1987
GRU	18.3897	6.9120	8.8052
LSTM	17.8518	7.0439	9.0199
DWT-LSTM	11.5884	4.3377	5.9291

## Data Availability

Raw data used to support the results of this study are included in the article.

## References

[1] Rijnders E., Janssen N. A. H., Van Vliet P. H. N., Brunekreef B. (2001). Personal and outdoor nitrogen dioxide concentrations in relation to degree of urbanization and traffic density. *Environmental Health Perspectives*.

[2] Pandey J. S., Kumar R., Devotta S. (2005). Health risks of NO_2_, SPM and SO_2_ in Delhi (India). *Atmospheric Environment*.

[3] Chai T., Carmichael G. R., Tang Y. (2009). Regional NOx emission inversion through a four-dimensional variational approach using SCIAMACHY tropospheric NO_2_ column observations. *Atmospheric Environment*.

[4] Han K. M., Song C. H., Ahn H. J. (2009). Investigation of NOx emissions and NOx-related chemistry in East Asia using CMAQ-predicted and GOME-derived NO_2_ columns. *Atmospheric Chemistry and Physics*.

[5] Wang L., Zhang Y., Wang K., Zheng B., Zhang Q., Wei W. (2016). Application of weather research and forecasting model with chemistry (WRF/Chem) over northern China: sensitivity study, comparative evaluation, and policy implications. *Atmospheric Environment*.

[6] Wang D., Jiang B., Li F., Lin W. (2018). Investigation of the air pollution event in Beijing-Tianjin-Hebei region in December 2016 using WRF-chem. *Advances in Meteorology*.

[7] Baykara M., Im U., Unal A. (2019). Evaluation of impact of residential heating on air quality of megacity Istanbul by CMAQ. *Science of the Total Environment*.

[8] Hinojosa-Baliño I., Infante-Vázquez O., Vallejo M. (2019). Distribution of PM2.5 air pollution in Mexico city: spatial analysis with land-use regression model. *Applied Sciences*.

[9] Alahmadi S., Al-Ahmadi K., Almeshari M. (2019). Spatial variation in the association between NO_2_ concentrations and shipping emissions in the Red Sea. *Science of the Total Environment*.

[10] Mirzaei M., Amanollahi J., Tzanis C. G. (2019). Evaluation of linear, nonlinear, and hybrid models for predicting PM 2.5 based on a GTWR model and MODIS AOD data. *Air Quality, Atmosphere & Health*.

[11] Zhao M., Li X. An application of spatial decision tree for classification of air pollution index.

[12] Gutiérrez L., Mena R. H., Ruggiero M. (2016). A time dependent Bayesian nonparametric model for air quality analysis. *Computational Statistics & Data Analysis*.

[13] Oprea M., Popescu M., Mihalache S. F. A neural network based model for PM 2.5 air pollutant forecasting.

[14] Masih A. Application of random forest algorithm to predict the atmospheric concentration of NO2.

[15] Liu B. C., Binaykia A., Chang P. C., Tiwari M. K., Tsao C. C. (2017). Urban air quality forecasting based on multi-dimensional collaborative support vector regression (SVR): a case study of Beijing-Tianjin-Shijiazhuang. *PLoS One*.

[16] Cabaneros S. M. S., Calautit J. K. S., Hughes B. R. (2017). Hybrid artificial neural network models for effective prediction and mitigation of urban roadside NO_2_ pollution. *Energy Procedia*.

[17] Mishra D., Goyal P. (2016). Neuro-fuzzy approach to forecast NO_2_ pollutants addressed to air quality dispersion model over Delhi, India. *Aerosol and Air Quality Research*.

[18] Li X., Peng L., Yao X. (2017). Long short-term memory neural network for air pollutant concentration predictions: method development and evaluation. *Environmental Pollution*.

[19] Reddy V., Yedavalli P., Mohanty S., Nakhat U. (2018). Deep air: forecasting air pollution in Beijing, China. http://arxiv.org/abs/1804/1804.07891.

[20] Lu W. Z., Xue Y. (2014). Prediction of particulate matter at street level using artificial neural networks coupling with chaotic particle swarm optimization algorithm. *Building and Environment*.

[21] Ma J., Ding Y., Cheng J. C. P., Jiang F., Wan Z. (2019). A temporal-spatial interpolation and extrapolation method based on geographic Long Short-Term Memory neural network for PM2.5. *Journal of Cleaner Production*.

[22] Song X., Huang J., Song D. Air quality prediction based on LSTM-kalman model.

[23] Yuan W., Wang K., Bo X., Tang L., Wu J. (2019). A novel multi-factor & multi-scale method for PM2.5 concentration forecasting. *Environmental Pollution*.

[24] Huang C.-J., Kuo P.-H. (2018). A deep CNN-LSTM model for particulate matter (PM2.5) forecasting in smart cities. *Sensors*.

[25] Rezamand M., Kordestani M., Carriveau R., Ting D. S. K., Saif M. (2020). An integrated feature-based failure prognosis method for wind turbine bearings. *IEEE/ASME Transactions on Mechatronics*.

[26] Zhang D., Bailey A. D., Djurdjanovic D. (2016). Bayesian identification of hidden markov models and their use for condition-based monitoring. *IEEE Transactions on Reliability*.

[27] Kordestani M., Samadi M. F., Saif M. (2020). A new hybrid fault prognosis method for MFS systems based on distributed neural networks and recursive bayesian algorithm. *IEEE Systems Journal*.

[28] Rezamand M., Kordestani M., Orchard M., Carriveau R., Ting D. S. K., Saif M. (2020). Improved remaining useful life estimation of wind turbine drivetrain bearings under varying operating conditions (VOC). *IEEE Transactions on Industrail Informatic*.

[29] Chen C., Zhang B., Vachtsevanos G. (2012). Prediction of machine health condition using neuro-fuzzy and Bayesian algorithms. *IEEE Transactions on Instrumentation and Measurement*.

[30] Bai Y., Zeng B., Li C., Zhang J. (2019). An ensemble long short-term memory neural network for hourly PM2.5 concentration forecasting. *Chemosphere*.

[31] Zhao J., Deng F., Cai Y., Chen J. (2019). Long short-term memory—fully connected (LSTM-FC) neural network for PM2.5 concentration prediction. *Chemosphere*.

[32] Pak U., Kim C., Ryu U., Sok K., Pak S. (2018). A hybrid model based on convolutional neural networks and long short-term memory for ozone concentration prediction. *Air Quality, Atmosphere & Health*.

[33] Wu Q., Lin H. (2019). A novel optimal-hybrid model for daily air quality index prediction considering air pollutant factors. *Science of the Total Environment*.

[34] Xiao K., Wang Y., Wu G., Fu B., Zhu Y. (2018). Spatiotemporal characteristics of air pollutants (PM10, PM2.5, SO_2_, NO_2_, O_3_, and CO) in the inland basin city of Chengdu, southwest China. *Atmosphere*.

[35] Bai Y., Li Y., Zeng B., Li C., Zhang J. (2019). Hourly PM2.5 concentration forecast using stacked autoencoder model with emphasis on seasonality. *Journal of Cleaner Production*.

[36] Hochreiter S., Schmidhuber J. (1997). Long short-term memory. *Neural Computation*.

[37] Gers F. A., Schmidhuber J. Recurrent nets that time and count.

[38] Petersen N. C., Rodrigues F., Pereira F. C. (2019). Multi-output bus travel time prediction with convolutional LSTM neural network. *Expert Systems with Applications*.

[39] Kim H. Y., Won C. H. (2018). Forecasting the volatility of stock price index: a hybrid model integrating LSTM with multiple GARCH-type models. *Expert Systems with Applications*.

[40] Liu H., Mi X.-w., Li Y.-f. (2018). Wind speed forecasting method based on deep learning strategy using empirical wavelet transform, long short term memory neural network and Elman neural network. *Energy Conversion and Management*.

[41] Daubechies I. (1988). Orthonormal bases of compactly supported wavelets. *Communications on Pure and Applied Mathematics*.

[42] Ming H., Huang D., Xie L., Wu J., Dong M., Li H. (2016). Deep bidirectional LSTM modeling of timbre and prosody for emotional voice conversion. *Interspeech*.

[43] Sugiartawan P., Pulungan R., Sari A. K. (2017). Prediction by a hybrid of wavelet transform and long-short-term-memory neural network. *International Journal of Advanced Computer Science and Applications*.

[44] Li X., Peng L., Hu Y., Shao J., Chi T. (2016). Deep learning architecture for air quality predictions. *Environmental Science and Pollution Research*.

[45] Xu Y., Yang W., Wang J. (2017). Air quality early-warning system for cities in China. *Atmospheric Environment*.

[46] Yuan M., Wu Y., Lin L. Fault diagnosis and remaining useful life estimation of aero engine using LSTM neural network.

